# Performance of low-educated elders with depression on Addenbrooke's Cognitive Examination-Revised (ace-r) test

**DOI:** 10.1590/S1980-57642016DN10100004

**Published:** 2016

**Authors:** Michele Beckert, Fernanda Loureiro, Caroline Menta, Elisa Fasolin Mello, Eduardo L. Nogueira, Armin von Gunten, Irênio Gomes

**Affiliations:** 1 Psychologist, MD, Department of Geriatrics and Gerontology, Pontifical Catholic University of Rio Grande do Sul, RS, Brazil; 2 Speech Therapist, MD, PhD, Institute of Geriatrics and Gerontology, Pontifical Catholic University of Rio Grande do Sul, RS, Brazil; 3 Psychiatrist, MD, Institute of Geriatrics and Gerontology, Pontifical Catholic University of Rio Grande do Sul, RS, Brazil; 4 Psychiatrist, MD, Institute of Geriatrics and Gerontology, Pontifical Catholic University of Rio Grande do Sul, RS, Brazil; 5 Psychiatrist, MD, PhD, Institute of Geriatrics and Gerontology, Pontifical Catholic University of Rio Grande do Sul, RS, Brazil; 6 Psychiatrist, MD, Institute of Geriatrics and Gerontology, Pontifical Catholic University of Rio Grande do Sul, RS, Brazil, and Old-Age Psychiatric Service, Department of Psychiatry, Lausanne University Hospital, Switzerland; 7 Neurologist, MD, PhD, Institute of Geriatrics and Gerontology, Pontifical Catholic University of Rio Grande do Sul, RS, Brazil

**Keywords:** cognitive evaluation, depression, elderly, low education, ACE-R, avaliação cognitiva, depressão, idoso, baixa escolaridade, ACE-R

## Abstract

Along with cognitive disorders, depression has been a concern for mental health services due to its highly debilitating effect on the functioning and quality of life of the elderly. However, there is still little understanding of the cognitive alterations resulting from depression or of the difficult differential diagnosis with mild cognitive impairment (MCI). It is known that performance on cognitive tests is strongly influenced by education but few studies have been conducted involving low-educated populations.

**Objective:**

To evaluate the performance of elders with low education and no dementia on Addenbrooke's Cognitive Examination-Revised (ACE-R) test and its cognitive domains, and compare patients with Current Major Depressive Episode (CMDE) against those without depressive symptoms.

**Methods:**

A retrospective, cross-sectional analytical study was conducted based on medical files of patients treated at the Cerebral Aging Clinic of the Hospital São Lucas of the PUCRS. The study included 116 individuals with low education (< 8 years of education) aged between 60 and 84 (69.6 ± 6.4) years, with MCDE (N = 41) and controls (N = 75).

**Results:**

No significant difference was observed between control and MCDE groups in median scores on the ACE-R, Mini-Mental State Examination, and the five cognitive domains. There was also no difference between the groups on separate analyses of results on the clock drawing test, the categorical verbal and phonological fluency test, and the naming test.

**Conclusion:**

The results of this study showed that depressive symptoms did not influence scores on the ACE-R tests conducted in elders with low education.

## RESUMO

Depressão, juntamente com os transtornos cognitivos, tem sido uma preocupação entre os serviços de saúde mental, devido ao alto índice de prejuízo na funcionalidade e qualidade de vida desta população. Contudo, ainda permanece em aberto a compreensão das alterações cognitivas decorrentes da depressão e de difícil diagnóstico diferencial com o comprometimento cognitivo leve (CCL). Sabe-se que o desempenho nos testes cognitivos é fortemente influenciados pela escolaridade, no entanto, poucos estudos tem sido realizados em populações de muito baixa escolaridade.

**Objetivo:**

Avaliar o desempenho no Addenbrooke's Cognitive Examination-Revised (ACE-R) e seus domínios cognitivos em idosos de baixa escolaridade, sem demência, e comparar aqueles que tem diagnóstico de Episódio de Depressão Maior Atual (EDMA), com os que não têm quadro depressivo.

**Métodos:**

Estudo transversal analítico, retrospectivo, através dos prontuários dos pacientes atendidos no Ambulatório de Envelhecimento Cerebral (AMBEC) do Hospital São Lucas da PUCRS. Foram incluídos 116 indivíduos com baixa escolaridade (< 8 anos de estudo) e idade entre 60 e 84 anos (69,6 ± 6,7), com EDMA (N = 41) e controles (N = 75).

**Resultados:**

Na comparação das médias do ACE-R e os cinco domínios cognitivos, entre o grupo controle e o grupo com EDMA, não foi observada diferença significativa. Também não houve diferença entre os grupos quando analisado separadamente os resultados do teste do relógio, da fluência verbal categórica e fonológica e do teste de nomeação.

**Conclusão:**

Como observado neste estudo, os sintomas depressivos não modificam os valores dos testes realizados no ACE-R de idosos com baixa escolaridade.

## INTRODUCTION

Depression is one of the most prevalent^1^^,^ 2 psychiatric disorder among the elderly and has a huge impact on functional capacity^3^ and quality of life. Furthermore, 40-60% of depressed patients without dementia have cognitive deficits.^4^ However, our understanding of the cognitive impairment resulting from depression remains incomplete.

Herrmann et al. (2013)^5^ showed that a proportion of elderly patients with depression exhibit impairments in processing time, episodic memory, executive function and semantic memory. In addition, changes in visual-spatial abilities, working memory and attention and inhibition control have also been described (O'Hara et al., 2006).^6^ Cognitive evaluation is influenced by cultural aspects, socio-economic conditions and educational level.^7^ There are some studies on the elderly with low education.^7^^,^ 8 Currently, few cognitive assessment instruments are available that consider normalcy levels for illiterate individuals.

The tests normally used in cognitive screening, such as the Mini-Mental State Examination (MMSE)^9^ and the Clock Drawing^10^ and Verbal Fluency^11^ tests have good sensitivity and specificity for early diagnosis. However, performance on these tests is highly influenced by education,^12^ and populations in low and middle income countries perform differently from those observed in high income countries. Several studies have sought to better understand and define specific criteria for these situations.^13^^,^ 14 Scant studies have been conducted in populations with very low education (illiterate or individuals with less than 4 years of education)^15^^,^ 16 and even fewer on how their cognitive performance is influenced by depressive symptoms.

Addenbrooke's Cognitive Examination - Revised (ACE-R) is a global cognitive evaluation instrument that includes the MMSE score as well as additional memory and naming evaluations and fluency and clock tests. This provides an examination with higher sensitivity for the initial symptoms of cognitive decline and with specific sub-scores for cognitive domains.^17^ When comparing ACE-R performance of patients with cognitive complaints due to dementia, emotional changes, or a combination of the two, the total score for the group with depression was no different from the control group. However, when each cognitive area was analyzed separately, the tests for memory and phonological fluency showed a significantly lower score among depressed subjects.^18^

The aim of this study was to compare the ACE-R score and its sub-tests between persons with diagnosis of Current Major Depressive Episode (CMDE) and those without symptoms of depression, in elders with low education and no dementia.

## METHODS

**Study outline and population.** This was a cross-sectional study based on analysis of medical records collected retrospectively from patients treated at the Cerebral Aging Clinic (CAC) of the Hospital São Lucas of PUCRS. The CAC was created in partnership with the Porto Alegre Family Health Strategy with the goal of assessing the mental health of the elderly by treating patients identified by Community Health Agents as having potential cognitive decline or depressive symptoms. These elderly patients underwent clinical evaluation performed by a neurologist and a psychiatrist, with many of those evaluated showing no neurological or psychiatric disorders.

The study included 116 individuals with low education (< 8 years of schooling) between 60 and 84 years of age (69.6 ± 6.7) assessed at the CAC between April 2012 and September 2014. Patients were classified as those with a CMDE diagnosis (N = 41) and controls without depression (N = 75). The subjects had no clinical diagnosis of MCI or dementia. Patients with structural or degenerative diseases of the central nervous system including stroke, brain tumors, axis I psychiatric disorders such as bipolar disorder or schizophrenia, or a history of current or past alcohol or drug abuse and undercorrected auditory or visual difficulties were also excluded. 

**Data collection.** The routine evaluation at the CAC is based on a multi-professional team of psychiatrists, neurologists and neuropsychologists that evaluated the patients. The psychiatric evaluation was conducted based on the application of the Geriatric Depression Scale (GDS)^19^^,^ 20 and the MINI PLUS^21^ version 5 by psychiatrists previously trained on these instruments. The criteria of the Diagnostic and Statistical Manual of Mental Disorders - DSM-IV-TR^22^ were used for the CMDE diagnosis. The neurological evaluation comprised a clinical evaluation (history taking and physical exam) and the application of the ACE-R which also includes the MMSE.^18^ The criteria recommended by the National Institute on Aging-Alzheimer's Association^23^^,^ 24 were used to clinically diagnose MCI and dementia. In this evaluation, MMSE scores were taken into consideration as indicators of objective cognitive impairment, although the final score was not used as the defining diagnostic criteria. A complementary neuropsychological evaluation was performed when necessary. The cut-off points of the MMSE used in our clinic as suggestive of cognitive decline are 17 for the illiterate or functional illiteracy and 22 for those with 1-7 years of education, as described by Laks et al. for the Brazilian population.^25^ There is no defined cut-off point for a Brazilian population with low education for the ACE-R. The score of 65 can be considered as a reference for defining normalcy only for individuals with 4-7 years of education, based on reports for the Brazilian population, a score which represents two standard deviations below the average.^18^ The final diagnostic definition was based on the clinical criteria, using information provided by the informant and from subsequent visits.

The present study was approved by the PUCRS Ethics and Research Committee, under document number 933.235 according to Resolution 466/12, and the study authors signed a confidentiality agreement in order to use the data.

**Data analysis.** The ACE-R evaluates attention and orientation (score 0-18), memory (0-26), phonological and semantic verbal fluency (0-14), language (0-26), visual-spatial ability (0-16), giving a total of 100 points. Besides these functions, the clock drawing test, semantic and phonological fluency, and naming were analyzed separately. The score used for the clock drawing lies in the 0-5 range, according to the ACE-R scoring instructions (circle = 1, numbers = 2, hands = 2). The total number of words recalled in one minute was used as the raw score for semantic (animal names generated in 1 minute) and phonological (words generated beginning with the letter P in 1 minute) fluency. Naming score was based on number of correct answers in naming the 12 figures featured in the ACE-R.

The data was stored in a database designed specifically for this project using FileMaker Pro 12 and analyzed with the aid of the statistical package SPSS 17. Data was described as absolute and relative frequencies, means and standard deviations. Comparison of demographic characteristics between the groups was performed using Pearson's Chi-squared test. The means of the cognitive evaluation scores of each group were compared using Student's t-test. A multiple linear regression model was used to compare the groups, adjusted for age, education and gender. The Pearson correlation test was applied to the results of 94 individuals to verify possible associations between the GDS-15 and the ACE-R cognitive domains (n = 94). The significance level considered was P < 0.05.

## RESULTS

Of the 116 individuals studied, 83% were female, 13% over 80 years old, and 36% illiterate. A total of 41 patients were diagnosed with CMDE, with a higher prevalence among women (Table 1). Median age was higher in the non-depressed group (70.7 ± 6.8) than in the group with CMDE (67.5 ± 6.5).

**Table 1.** Distribution of demographic characteristics and prevalence of current major depressive episode according to the characteristics in 116 elders treated at the reference clinic of a mental health program under the Family Health Strategy in Porto Alegre-RS, Brazil.

**Variable**		**Population distribution n (%)**	CMDE*	
			**PREV^+^ (%)**	**P^§^**
Gender	Male	33 (28.4)	9.1	<0.001
	Female	83 (71.6)	45.8	
Age group	60-69	63 (54.3)	42.9	0.074
	70-79	40 (34.5)	27.5	
	80 or +	13 (11.2)	23.1	
Education	Illiterate	36 (31.0)	36.1	0.956
	1-3 years	32 (27.6)	34.4	
	4-7 years	48 (41.4)	35.4	
Total		116 (100)	35.3	

*Current Major Depressive Episode. +Prevalence. §P Value calculated by Pearson's Chi-squared test for gender variable and by Chi-squared linear tendency test for other variables.

No significant difference was observed between the control and depressed groups on comparisons of the median results of the different cognitive ACE-R domains, after adjusting for age and gender. There was also no difference between the groups when separately analyzing the results of the clock drawing test, categorical and phonological verbal fluency, and the naming test (Table 2).

**Table 2.** Median and standard deviation results on the ACE-R and its domains according to presence of Current Major Depressive Episode in 116 elders treated at the reference clinic of a mental health program under the Family Health Strategy of Porto Alegre-RS, Brazil.

**Variable**	**Depression**		**P^#^**	**P**
	**Yes m ± SD**	**No m ± SD**		
ACE-R Total*	65.95 ± 10.95	65.93 ± 11.86	0.829	0.977
ACE_AO^++^	14.58 ± 2.37	14.38 ± 2.26	0.615	0.445
ACE_MEM ^§^	15.05 ± 3.79	14.76 ± 4.55	0.651	0.601
ACE_FLU^||^	6.57 ± 2.62	7.27 ± 2.97	0.428	0.649
ACE_LANG^¶^	18.76 ± 4.18	18.53 ± 4.33	0.607	0.485
ACE_VISO^**^	11.10 ± 2.99	11.00 ± 2.76	0.753	0.965
FLU_ANI^+++^	11.87 ± 3.65	12.38 ± 4.06	0.873	0.957
FLU_FON^--^	7.76 ± 3.46	7.96 ± 4.08	0.819	0.749
CLOCK	3.28 ± 1.54	3.42 ± 1.27	0.726	0.476
NAM***	9.34 ± 2.34	9.62 ± 1.87	0.591	0.705

*Addenbrooke's Cognitive Examination. +Mini-Mental State Examination. ++Attention and Orientation subtest. §Memory subtest. ||Fluency subtest. ¶Language subtest. ** Visual-spatial ability subtest. +++Animals verbal fluency test. -phonological verbal fluency test (letter P). ***12 figures naming score on the ACE-R. #P calculated using Student's t-test for independent variables.

**Figure 1.**
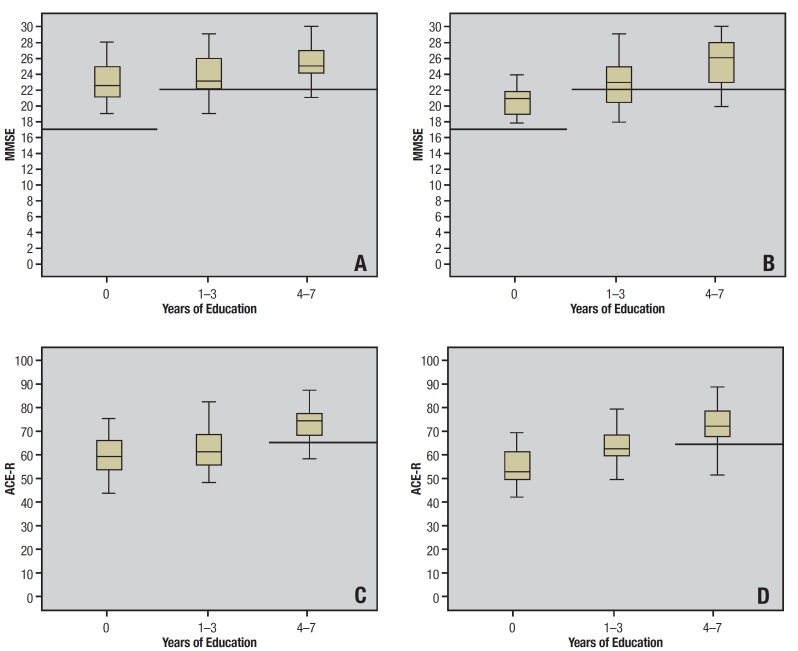
[A] Median MMSE performance of healthy group participants stratified by years of education. Cut-off point is 17 for illiterates and 22 for 1 to 7 years of education. [B] Median MMSE performance of CMDE group. [C] Median ACE-R test performance of healthy participants stratified by years of education. [D] Median ACE-R test performance of CMDE group stratified by years of education.

The median performance on the ACE-R and MMSE of both groups is shown in Figure 1. Low-educated individuals, with and without CMDE, performed slightly above the established cut-off points for this population, but well below the maximum score for each test. Median scores of the group of participants with 1-3 years of education o the ACE-R and MMSE were closest to the established cut-off points.

A second statistical analysis was used to verify the correlation between severity of depression measured using the score on the 15-item GDS and cognitive performance on the ACE-R and its domains. No correlation was found between the cognitive evaluation and severity of depressive symptoms (Table 3).

**Table 3.** Correlation test for ACE-R and its domains with Geriatric Depression Scale (GDS-15) in 91 elders treated at the reference clinic of a mental health program under the Family Health Strategy of Porto Alegre-RS, Brazil.

**Variable**	**R^#^**	**P**
ACE-R Total*	0.004	0.968
ACE_AO^+^	0.018	0.866
ACE_MEM^§^	-0.093	0.381
ACE_FLU^||^	-0.032	0.762
ACE_LANG^¶^	0.058	0.586
ACE_VISO^**^	0.060	0.573
FLU_ANI^+++^	-0.050	0.639
FLU_FON^--^	-0.093	0.476
CLOCK	0.097	0.362
NAM***	0.044	0.680

* Addenbrooke's Cognitive Examination. + Mini-Mental State Examination. ++ Attention and Orientation subtest. § Memory subtest. || Fluency subtest. ¶ Language subtest. ** Visual-spatial ability subtest. +++ Animals verbal fluency test. - phonological verbal fluency test (letter P). ***12 figures naming score on the ACE-R. # Pearson's correlation coefficient.

## DISCUSSION

This study found no difference in cognitive performance of low-educated elders diagnosed with CMDE when compared to the control group without depression. This was also observed on a separate analysis of cognitive domains and the tests comprising the naming task, categorical and phonological verbal fluency, and clock drawing assessments.

Executive functions are among the cognitive functions most sensitive to depression and the aging process.^26^ These functions are evaluated on the ACE-R through verbal fluency in the subtests for phonological fluency and categorical fluency and the clock-drawing test. Executive functions are considered to be complex constructs; therefore the phonological and categorical verbal fluency tests and the clock-drawing test were analyzed separately in order to interpret the performance of both groups for executive function. However, CMDE diagnosis had no impact on executive performance. The cut-off point proposed by Caramelli et al. (2007)^11^ for semantic fluency is 9 words in illiterate individuals and 12 words in those with 1-7 years of education. In the present study, the average number of words recalled by the depressed group was no different from the average recalled by the control group. Thus, depression at the time of the cognitive evaluation did not influence the categorical verbal fluency task in individuals with low education.

The clock drawing test is acknowledged to be an appropriate screening test for detecting dementia.^28^ In this study, no difference in performance on the clock drawing test between the study groups was found, showing no distinction between low-educated patients with and without depression. However, the performance on this test in both the healthy and depressed groups was well below the maximum score expected and must be interpreted with caution, especially for illiterate individuals. Kim and Chey (2010)^12^ found errors made on this test by illiterates to be similar to those of patients with Alzheimer's disease. According to these authors, semantic and visual-spatial representations and constructional abilities are necessary to translate the mental representation of a clock into a drawing, and the development of these abilities in illiterate individuals may have been deficient. Moon and Chey (2004)^29^ postulated that even when semantic and visual-spatial functions are intact, constructional abilities may be poorly developed in individuals with less education.

Naming performance was slightly superior in the elderly with depression. This findings contrasts with the usual observation of decreased naming and verbal fluency performance in depressed elderly (Novaretti et al. (2011).^30^ However, this decrease was not observed in the low educated sample. The depressed group was on average younger, but the statistical analysis was adjusted for age and gender.

In the study population, as can be seen in Figure 1, performances of the very low educated groups (1-3 years) with and without depression were very close to the established cut-off points for dementia and well below the maximum possible score on both the MMSE and the ACE-R. We believe that, as suggested by Manly et al. (2005),^31^ individuals with low education should be evaluated based not only on years of education but also that their reading and writing abilities be better analyzed to distinguish those who are functional illiterates, thus establishing more adequate cut-off points for their level of education.

In order to verify the influence of the severity of depression symptoms on cognitive performance, Pearson's correlation test was performed between the GDS-15 depression scale and the total score of the ACE-R and each of its cognitive domains. Again, the results showed no association between a worsening of depression symptoms and more severe cognitive impairment in individuals with low education. Depression may lower cognitive reserve. However, contrary to expectations, those with more cognitive reserve showed greater decline in cognitive performance as depressive symptoms increased than those with less cognitive reserve.^32^ These results indicate that the association between symptoms of depression and cognitive aspects of elders varies with level of cognitive reserve. While depressed individuals with high educational level experienced deterioration in their performance in memory, executive function and language tasks, individuals with low education maintained the same cognitive performance during increases on the depression scale. This is in keeping with the findings of our study, as increasing depressive symptoms had no influence on cognitive performance in individuals with low education. Similarly, a longitudinal study showed that symptoms of depression are associated with an increased risk of cognitive decline only in those with high levels of education.^33^

Patients with extremely low educational levels and illiterates are often not included in study samples due to the pencil and paper tasks related to reading and writing that are a part of cognitive testing. This study presents data that is important to further understanding of the cognitive performance of this population, and there is a need for more research on their normalcy levels as well as healthy and pathological cognitive aging. The main limitations of the present study are the fact that it is a cross-sectional study and used a convenience sample. We believe, however, that the characteristics of the outpatient clinic, to which elderly patients were referred after triage by the ACS as opposed to being sought by patient necessity, meant the population studied met the proposed objectives. Our findings suggest that, unlike elders with higher education, depressive symptoms in elders with low education does not influence cognitive performance on the ACE-R test. This shows that despite the presence of symptoms of depression, for an elderly population with less than 8 years of education or illiteracy, lower-than-expected ACE-R test scores may be due to true cognitive decline.
